# Heat Transfer Analysis for Stationary Boundary Layer Slip Flow of a Power-Law Fluid in a Darcy Porous Medium with Plate Suction/Injection

**DOI:** 10.1371/journal.pone.0138855

**Published:** 2015-09-25

**Authors:** Asim Aziz, Yasir Ali, Taha Aziz, J. I. Siddique

**Affiliations:** 1 College of Electrical and Mechanical Engineering, National University of Sciences and Technology, Rawalpindi, 46000, Pakistan; 2 DST-NRF Centre of Excellence in Mathematical and Statistical Sciences, University of the Witwatersrand, Wits 2050, South Africa; 3 Department of Mathematics, Pennsylvania State University, York Campus, 1031 Edgecomb Avenue, York, PA 17403, United States of America; North China Electric Power University, CHINA

## Abstract

In this paper, we investigate the slip effects on the boundary layer flow and heat transfer characteristics of a power-law fluid past a porous flat plate embedded in the Darcy type porous medium. The nonlinear coupled system of partial differential equations governing the flow and heat transfer of a power-law fluid is transformed into a system of nonlinear coupled ordinary differential equations by applying a suitable similarity transformation. The resulting system of ordinary differential equations is solved numerically using Matlab bvp4c solver. Numerical results are presented in the form of graphs and the effects of the power-law index, velocity and thermal slip parameters, permeability parameter, suction/injection parameter on the velocity and temperature profiles are examined.

## Introduction

The boundary layer flow play a vital role in many aspects of fluid mechanics and has been studied extensively for decades. A comprehensive literature survey on boundary layer theory and related topics can be found in the studies of [1–5]. Recent development in boundary layer models incorporate the analysis of heat transfer characteristics in a porous media because such processes exist in nature and have many engineering applications. Examples include but are not limited to heat exchanger, recovery of petroleum resources, fault zones, catalytic reactors, cooling devices, chemical reactions in a reactor chamber consisting of rectangular ducts, deposition of chemical vapor on surfaces and so on. A list of the key references in the vast literature concerning this field are given in [6–13].

It is revealed from the available literature on boundary layer models including heat transfer analysis that these are limited to Newtonian and some non-Newtonian fluids flow with traditional no-slip flow boundary conditions. Researchers extensively studied flow models for different geometries influenced by a number of factors including fluid viscosity, bounding surface characteristics, and external forces, to name a few. Limited attention was given to the slip boundary condition. Beavers and Joseph [14] first proposed a slip flow condition at the boundary. Since then, there has been a revival of interest in flow problems with slip conditions (see for example, [15–24]).

There are many models proposed for the non-Newtonian fluids. The theory of boundary layer for each proposed model is also available in the literature. It is beyond the scope of this work to revisit the vast amount of literature on the boundary layer flow of different non-Newtonian fluid models. Limited work on the topic can be referred as examples in [25–31]. Non-Newtonian fluids classified as either pseudoplastic or dilatant have become more common in industry as well as that of the analysis of the boundary layer flow and heat transfer characteristics. Dilatant or shear thickening fluids are liquids in which viscosity increases as the applied stress increases, whereas pseudoplastics or shear thinning fluids are characterized by the opposite relationship between viscosity and applied stress. The study of these so called power-law fluids is now a priority. One of the earliest studies on boundary layer flow of power-law fluids was made by Acrivos et al. [32] and Schowalter [33]. Lee and Ames [34] extended the above work to find the similarity solutions for power-law fluids. Andersson et al. [35] examined the boundary layer flow of electrically conducting power-law fluid in the presence of transverse of magnetic field. Recent additions considering flow of non-Newtonian power-law fluid with heat and mass transfer under different physical situations are given in [36–41].

In the present paper, we extend the work of [22] and investigate the slip effect on boundary layer flow of power-law fluid including heat transfer over a porous flat sheet embedded in a porous medium. The velocity and thermal slip conditions are taken in terms of shear stress. The similarity transformation approach is employed to transform the governing system of partial differential equations to a system of ordinary differential equations together with the boundary conditions. The resulting system of ordinary differential equations is solved numerically using the Matlab bvp4c solver. The results are shown in the form of graphs and discussed from a physical point of view.

## Problem Statement and Mathematical Formulation

**Fig 1:**
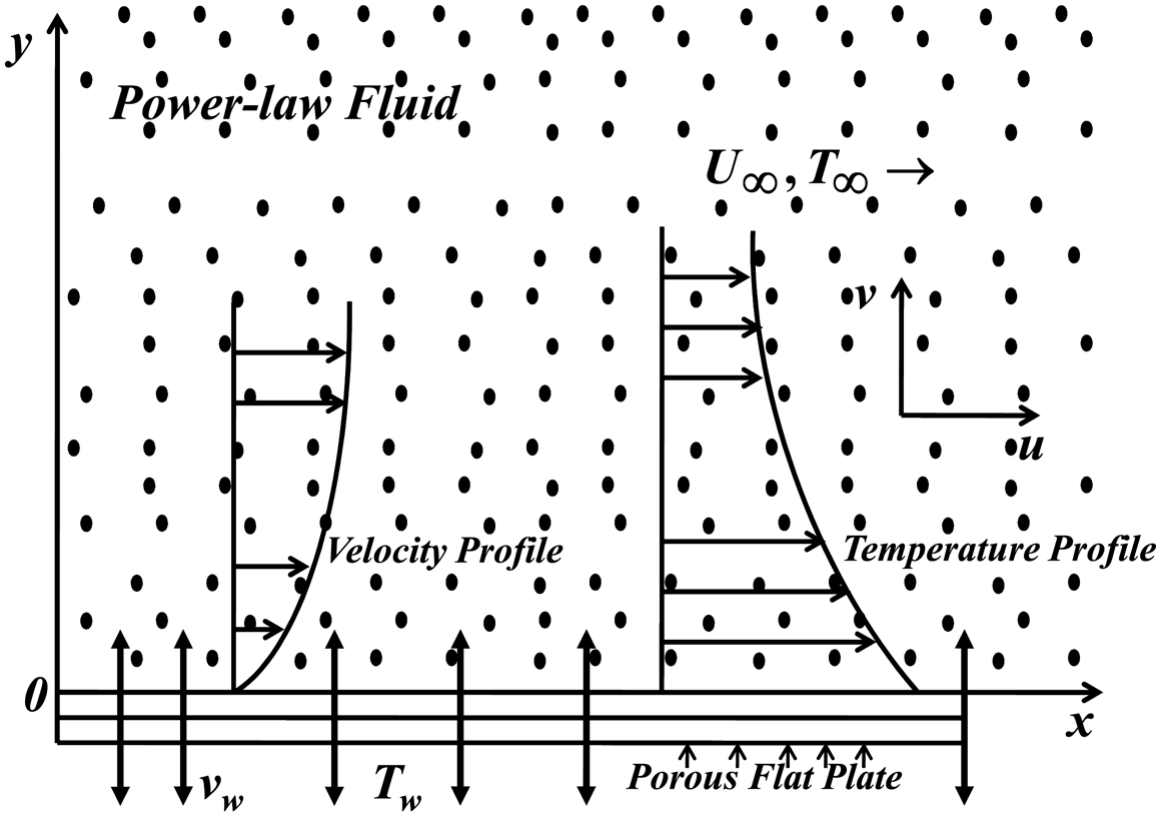
Schematic representation of geometry.

We consider the steady, two-dimensional flow and heat transfer of a power-law fluid over a semi-infinite porous plate in a porous medium. The surface of the plate is insulated and admits partial slip conditions. The leading edge of the plate is at *x* = 0. The plate coincides with the plane *y* = 0. The corresponding velocity components in the *x* and *y* directions are *u* and *v* respectively. The temperature of the plate is *T*_*w*_. The flow far away from the plate is uniform and in the direction parallel to the plate. The velocity and temperature far away from the plate are *U*_∞_ and *T*_∞_ respectively. The geometry of the flow model is given in Fig 1. Using the boundary layer approximations, the continuity, momentum and energy equations are written in usual notation as

$$\frac{\partial u}{\partial x}+\frac{\partial v}{\partial y}=0,$$ (1)

$$u\frac{\partial u}{\partial x}+v\frac{\partial u}{\partial y}=\frac{1}{\rho }\frac{\partial \tau_{xy}}{\partial y}-\frac{1}{\rho k}\left(u-U_{\infty }\right),$$ (2)

$$u\frac{\partial T}{\partial x}+v\frac{\partial T}{\partial y}=\frac{k}{\rho C_{p}}\frac{\partial^{2}T}{\partial y^{2}},$$ (3)

where *ρ* is the fluid density, *τ*_*xy*_ is the shear stress, *T* is temperature, *C*_*p*_ is the specific heat at constant pressure and *k* is the thermal conductivity of the fluid. The boundary conditions for the velocity and temperature fields are:

$$u=L_{1}(\frac{\partial u}{\partial y}),\;v=v_{w},\;T=T_{w}+D_{1}\;(\frac{\partial T}{\partial y})\;\text{at}\;y=0,$$ (4)

$$u\to U_{\infty },\;T\to T_{\infty }\;\text{as}\;y\to \infty .$$ (5)

In Eq (4), $L_{1}=L{\left(Re_{x}\right)}^{\frac{1}{2}}$ is the velocity slip factor and $D_{1}=D{\left(Re_{x}\right)}^{\frac{1}{2}}$ is the thermal slip factor with *L* and *D* are the initial values of velocity and thermal slip factors and have dimensions of length. Here $Re_{x}=\rho U_{\infty }^{2-n}x^{n}/K$ is the local Reynolds number and the velocity *v*_*w*_ defines suction or blowing through the porous plate.

The shear stress component *τ*_*xy*_ in Eq (2) for the power-law fluid model is defined as (see details in [29])

$$\tau_{xy}=K{\left|\frac{\partial u}{\partial y}\right|}^{n-1}\frac{\partial u}{\partial y},$$ (6)

where *K* is the consistency coefficient and *n* is the power-law index. In the above constitutive equation *n* = 1 corresponds to Newtonian fluid behavior. On the other hand, when *n* < 1 we observe the shear-thinning behavior of the fluid and for *n* > 1 shear-thickening behavior is observed.

Substitution of Eq (6) into Eq (2) result in

$$u\frac{\partial u}{\partial x}+v\frac{\partial u}{\partial y}=\frac{K}{\rho }\frac{\partial }{\partial y}({\left|\frac{\partial u}{\partial y}\right|}^{n-1}\frac{\partial u}{\partial y})-\frac{1}{\rho k}\left(u-U_{\infty }\right).$$ (7)

To obtain the dimensionless form of Eqs (3) and (7), we introduce the dimensionless similarity variable

$$\eta =\;{\left(\frac{Re}{x/L}\right)}^{\frac{1}{n+1}}\;\frac{y}{L},$$ (8)

where *L* is the characteristic length and $Re=\rho U_{\infty }^{2-n}L^{n}/K$ is the generalized Reynolds number. The dimensionless stream function *f*(*η*) and dimensionless temperature *θ*(*η*) are defined as

$$\psi \left(x,y\right)=LU_{\infty }{\left(\frac{x/L}{Re}\right)}^{\frac{1}{n+1}}f\left(\eta \right),\;\theta \left(\eta \right)=\frac{T-T_{\infty }}{T_{w}-T_{\infty }}.$$ (9)

The dimensionless stream function *ψ*(*x*,*y*) in Eq (9) identically satisfies the continuity Eq (1) with

$$u=\frac{\partial \psi }{\partial y},\;v=-\frac{\partial \psi }{\partial x}.$$ (10)

Introducing Eqs (8)–(10) into Eqs (1) and (7), we obtain the self-similar system of ordinary differential equations

$$n|f^{\prime \prime }{|}^{n-1}f^{{}^{\prime \prime \prime }}+\frac{1}{n+1}ff^{\prime \prime }-k^{*}f^{\prime }=0,$$ (11)

$$\theta^{\prime \prime }+\frac{1}{n+1}P_{r}f\theta^{\prime }=0,$$ (12)

where k* = 1/(*Da*_*x*_ *Re*_*x*_) represents the permeability of porous medium, *Da*_*x*_ = *k*_0_/*x* is the local Darcy number, *k*_0_ is a constant and $P_{r}={\left(\frac{U_{\infty }^{3}}{x}\right)}^{\frac{n-1}{n+1}}\frac{c_{p}\rho }{\kappa }{\left(\frac{K}{\rho }\right)}^{\frac{2}{n+1}}$ is a Prandtl number for the power-law fluid. The corresponding boundary conditions Eqs (4) and (5) takes the form

$$f\left(\eta \right)=S,\;f^{\prime }\left(\eta \right)=\delta f^{\prime \prime }\left(\eta \right)\;\theta \left(\eta \right)=1+\beta \theta^{\prime }\left(\eta \right)\;\text{at}\;\eta =0,$$ (13)

$$f^{\prime }\left(\eta \right)\to 1\;\theta \left(\eta \right)\to \infty \;\text{as}\;\eta \to \infty ,$$ (14)

with

$$S=\frac{-\left(n+1\right)v_{w}}{U_{\infty }}{\left(Re_{x}\right)}^{\frac{1}{2}},$$ (15)

represents suction/injection velocity at the plate for *v*_*w*_ < 0 and *v*_*w*_ > 0, respectively. Moreover, $\delta =L\frac{\rho U_{\infty }}{K}$ is the velocity slip parameter and $\beta =D\frac{\rho U_{\infty }}{K}$ is the thermal slip parameter.

## Method of Solution

The nonlinear coupled ordinary differential Eqs (11) and (12) together with boundary conditions Eqs (13) and (14) are solved numerically using the Matlab bvp4c solver. In order to use bvp4c, first we convert the ODEs Eqs (11) and (12) to a system of first order differential equations

$$f^{\prime }=p,\;p^{\prime }=q,\;q^{\prime }=-\frac{1}{n(n+1)}fq^{2-n}+k^{*}\left(p-1\right)q^{1-n},$$ (16)

$$\theta^{\prime }=z,\;z^{\prime }=-\frac{1}{n+1}P_{r}fz,$$ (17)

along with the boundary conditions

$$\begin{array}{c} f(\eta )=0,\;p(\eta )=\delta q(\eta ),\;at\;\eta =0;\;p(\eta )\to 1\;as\;\eta \to \infty \\ \;\theta (\eta )=1+\beta z(\eta ),\;at\;\eta =0;\;\theta (\eta )\to 0\;as\;\eta \to \infty . \end{array}$$ (18)

**Fig 2:**
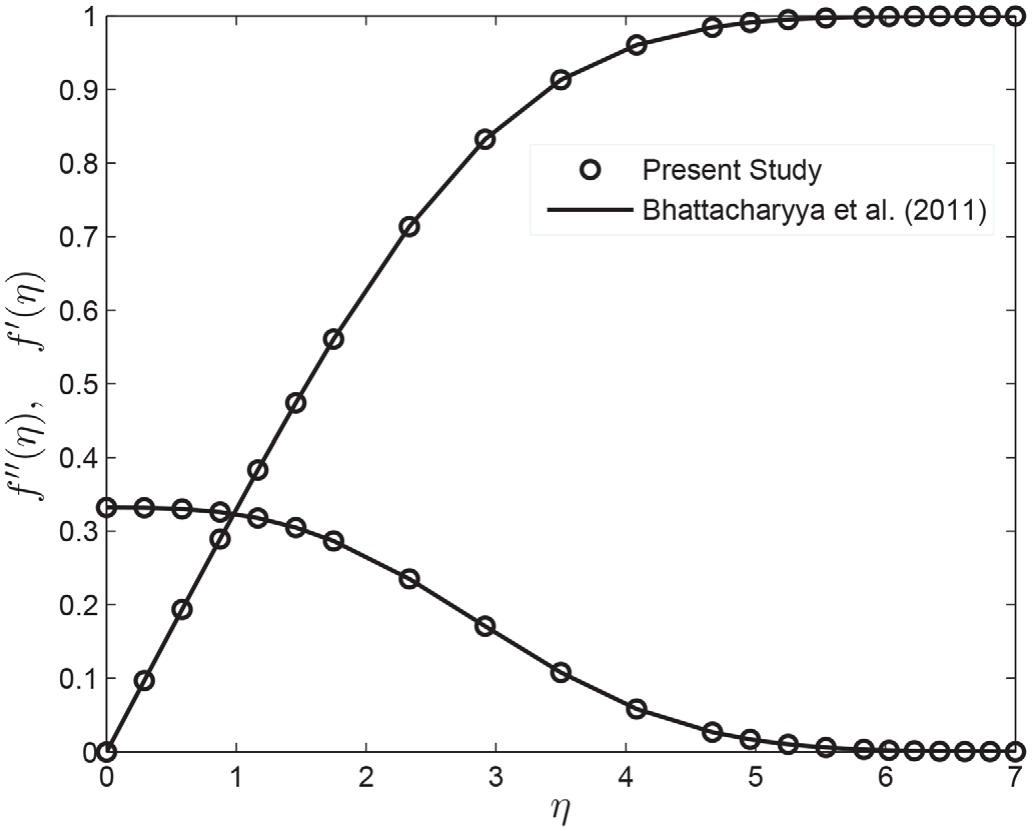
The velocity and shear stress profiles for power-law index *n* = 1, permeability parameter k* = 0 and velocity slip parameter *δ* = 0.

The bvp4c requires initial guesses for *q*(*η*) and *z*(*η*) at *η* = 0, and uses these initial values to generate solutions using a collocation method. In order to make an appropriate guess we start with a set of parameter values for which solution was known and progress until we obtain the solution of our problem. We verify the correctness of these solutions by comparing them with those obtained using the shooting method. We find the results to be in good agreement with the previous published results of [22] and [42] for *n* = 1 and k* = *δ* = 0 (see Fig 2).

In the next section, the numerical results are presented for the velocity and temperature functions of the coordinate *η*. The computations are performed for several values of the power-law index *n*, the velocity slip parameter *δ*, the thermal slip parameter *β*, the permeability parameter k* and the Prandtl number *Pr* and are depicted in Figs 3 to 11.

## Results and Discussion

**Fig 3:**
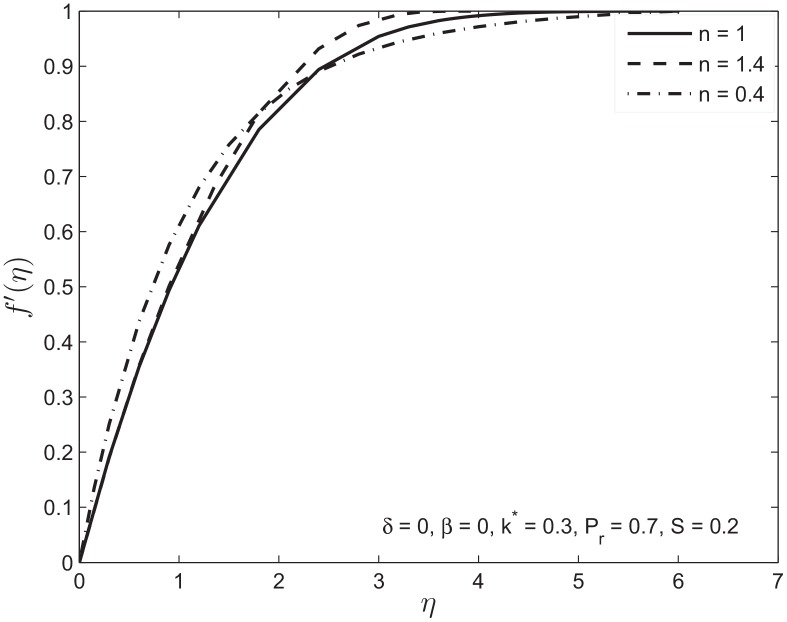
The velocity profiles *f*′(*η*) for power-law index *n* = 1, 0.4, 1.4.

**Fig 4:**
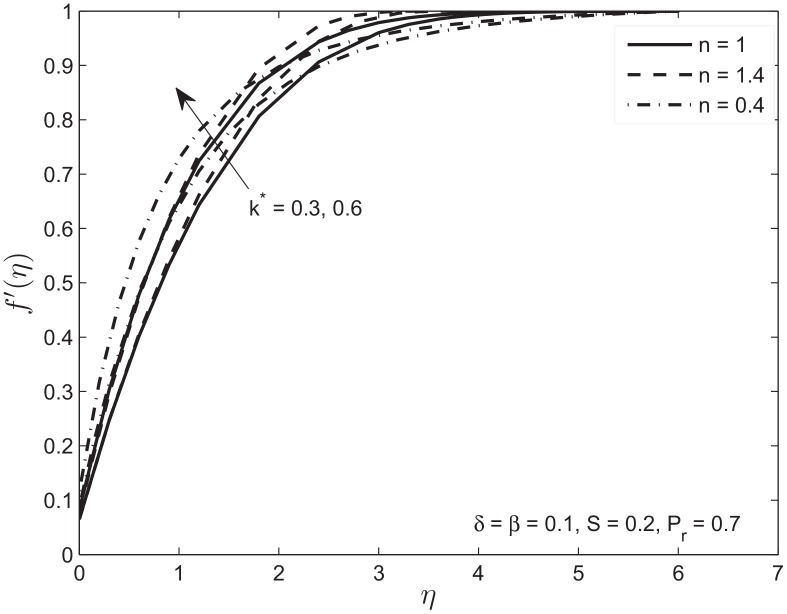
Effect of variation in permeability parameter k* on velocity profile *f*′(*η*) of Newtonian and non-Newtonian fluids.

Fig 3 illustrate the relative boundary layer thicknesses for Newtonian and non-Newtonian fluids with no-slip condition. Initially the shear-thinning fluid *n* < 1 rises faster than the shear-thickening fluid *n* > 1. This general observation is consistent with the recognition that at early times, when the strain rates are the largest, the shear-thinning fluid will have the smallest effective viscosity while the shear-thickening fluid will have the largest effective viscosity. Similarly, at later times, the strain rates get smaller and the effective viscosity of the shear thinning fluid will increase. Fig 4 shows the effect of permeability k* on the velocity profile with slip boundary conditions. It is observed that the velocity along the plate increases with increase in the permeability. This in turn decreases the thickness of the momentum boundary layer. This observation is consistent with recognition that an increase in the porosity of the medium decreases the magnitude of the Darcian body force which enhances the motion of the fluid in the boundary layer and ultimately decelerates the fluid particles in the porous medium.

Figs 3 and 4 with permeability k* = 0.3 gives variation of velocity profiles with slip and no-slip boundary conditions for the Newtonian and non-Newtonian fluids. Increase in velocity slip parameter increases the fluid velocity at the given distance from the plate. This in turn decreases the boundary layer thickness.

**Fig 5:**
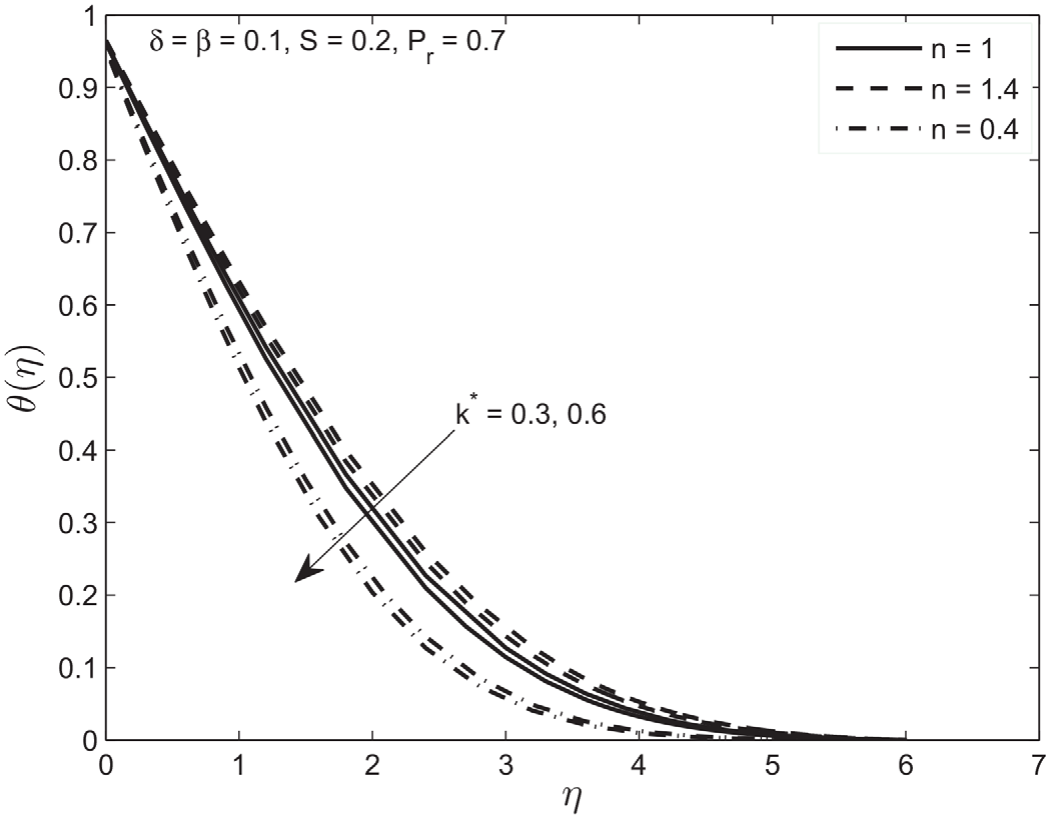
Effect of variation in permeability parameter k* on temperature profile *θ*(*η*) of Newtonian and non-Newtonian fluids.

The slip and permeability effect on the temperature profile *θ*(*η*) for both the Newtonian and non-Newtonian fluids are presented in Fig 5. The increase in permeability of the porous medium under slip conditions, decrease the temperature at any given point and the thermal boundary layer thickness. In other words the increase in permeability of porous medium decreases the thickness of momentum boundary layer which eventually increases the heat transfer. Furthermore, Fig 5 also illustrates the thermal boundary layer thickness for non-Newtonian fluids of different power-law exponents. A non-Newtonian fluid with a power-law exponent, 0.4 has relatively thin thermal boundary layer (*f* approaches zero quickly). However, if *n* = 1.4, the boundary layer is relatively thick.

**Fig 6:**
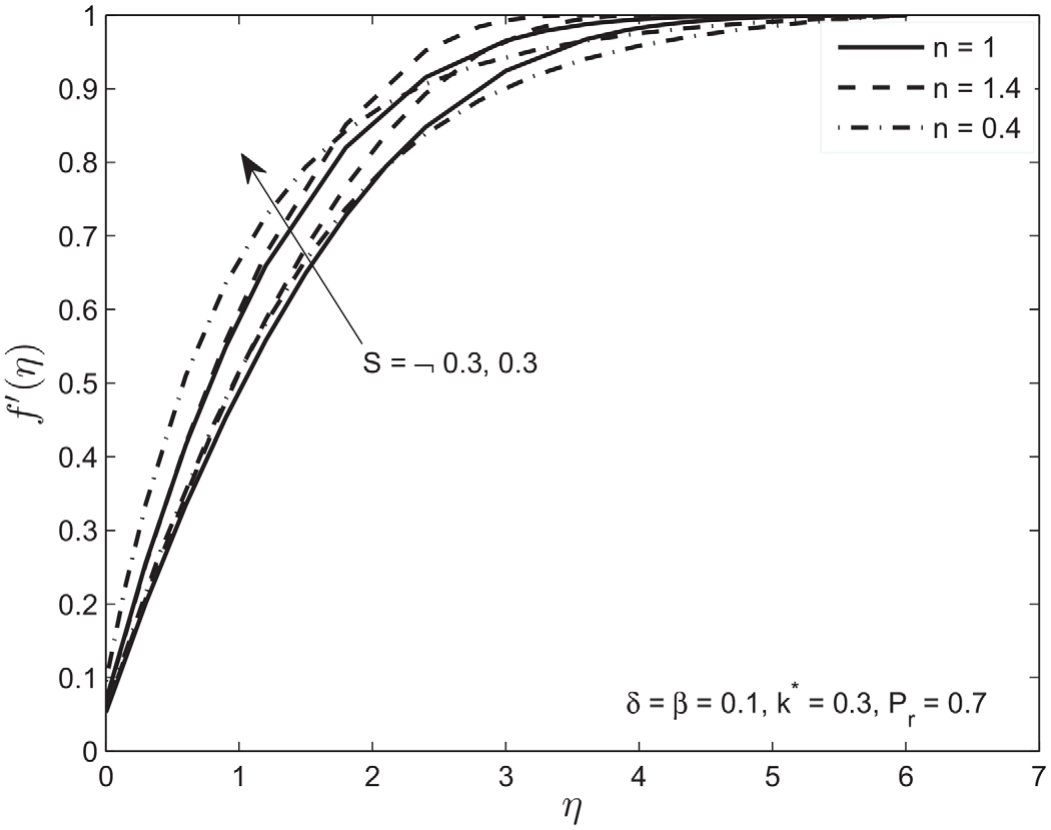
Effect of variation in suction/injection parameter *S* on velocity profile *f*′(*η*) of Newtonian and non-Newtonian fluids.

**Fig 7:**
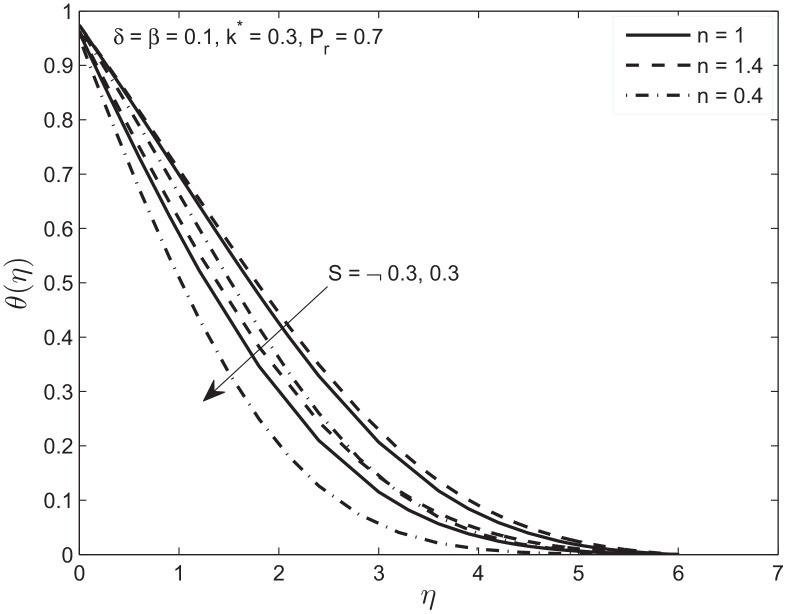
Effect of variation in suction/injection parameter *S* on temperature profile *θ*(*η*) of Newtonian and non-Newtonian fluids.

Figs 6 and 7 depict the effects of the suction (*S* > 0) and the injection (*S* < 0) parameter on the velocity and temperature profiles in the presence of slip conditions on the boundary of flat plate sandwiched in porous medium. In the case of suction, the momentum boundary layer decreases because at the wall fluid particles are drawn out. This eventually causes the increase in the velocity gradient as well as skin friction. This behavior is observed for both Newtonian and non-Newtonian fluids. Again we observe the velocity of the shear thinning fluid is faster in the beginning due to the above mentioned reason. For the case of injection an opposite trend is observed. Fig 7 shows the decrease in temperature *θ*(*η*) when suction parameter is increased. This causes a decrease in thermal boundary layer thickness by bringing the fluid closer to wall which in turn increases the rate of heat transfer. For the case of injection the increase in temperature profile is observed. Note that the decrease (*S* > 0) and increase (*S* < 0) in temperature profile is slower for shear thickening *n* > 1 compared to a Newtonian fluid and faster for shear thinning fluid *n* < 1.

**Fig 8:**
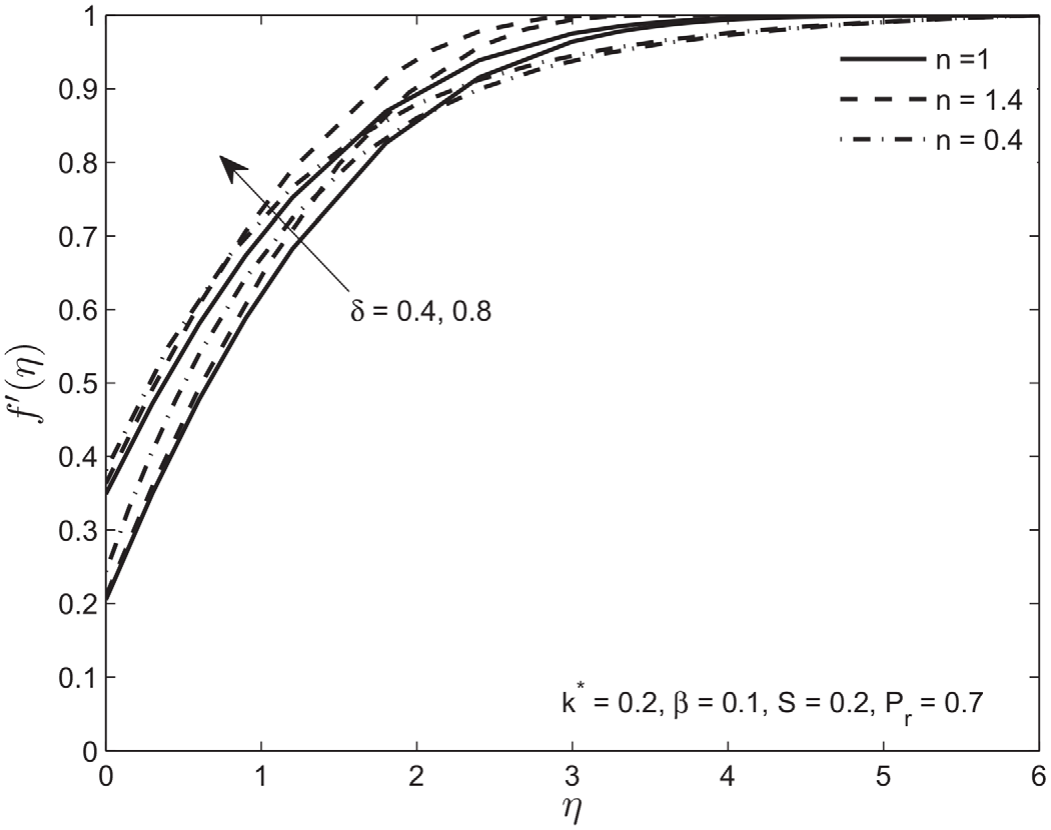
Effect of variation in velocity slip parameter *δ* on velocity profile *f*′(*η*) of Newtonian and non-Newtonian fluids.

**Fig 9:**
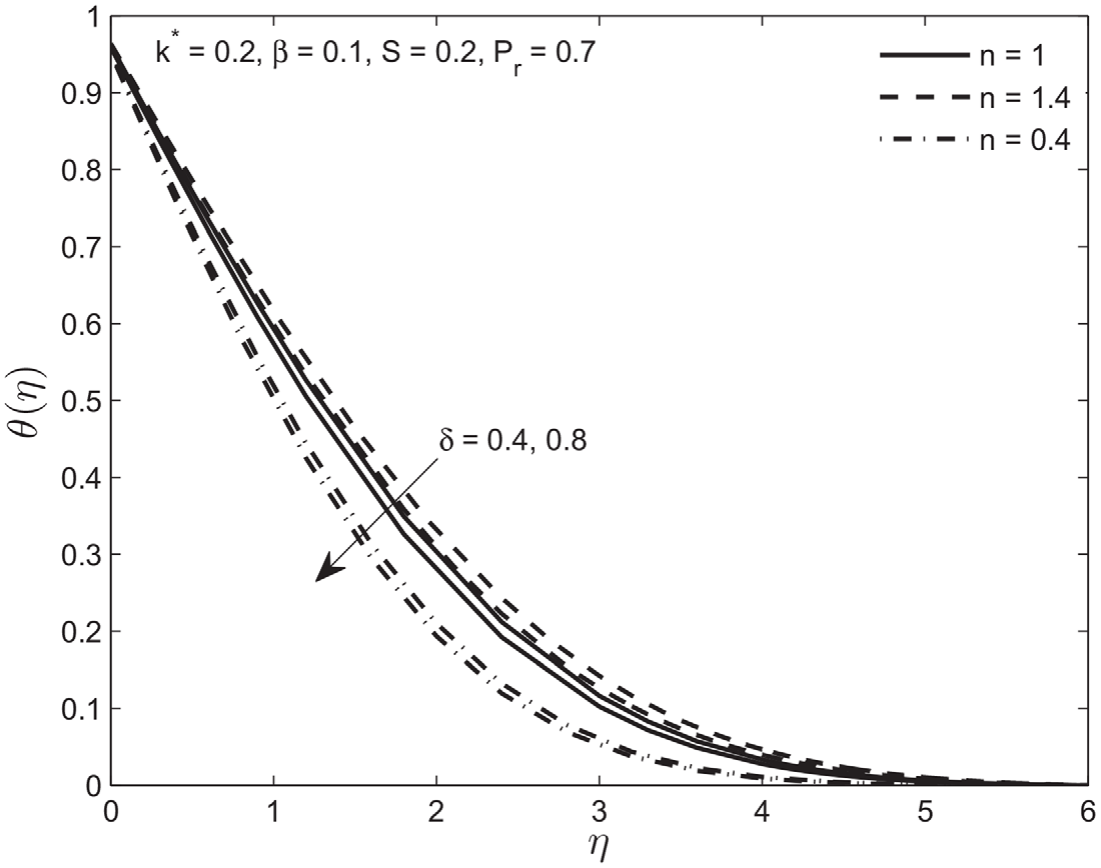
Effect of variation in velocity slip parameter *δ* on temperature profile *θ*(*η*) of Newtonian and non-Newtonian fluids.

Below we show the influence of the velocity slip parameter on the velocity and temperature profiles. In Fig 8 we used two values of velocity slip parameter for Newtonian, shear thickening, and shear thinning fluids. We observe that increase in slip parameter reduces the boundary layer thickness because of positive values of the fluid velocity at surface of the plate. This trend is slower in shear-thickening fluid and faster in shear-thinning fluid as compared to a Newtonian fluid. In Fig 9 the temperature profile *θ*(*η*) is plotted for different value of thermal slip parameter *δ*. The increase in temperature slip parameter reduces the temperature of power law fluid for a given distance from the porous plate. The temperature profile for shear-thickening fluid decreases slower than the Newtonian fluid. The opposite trend is observed for the shear-thinning fluid for increase in the slip parameter. In each case increase in velocity due to slip parameter is responsible for the increase in heat transfer.

**Fig 10:**
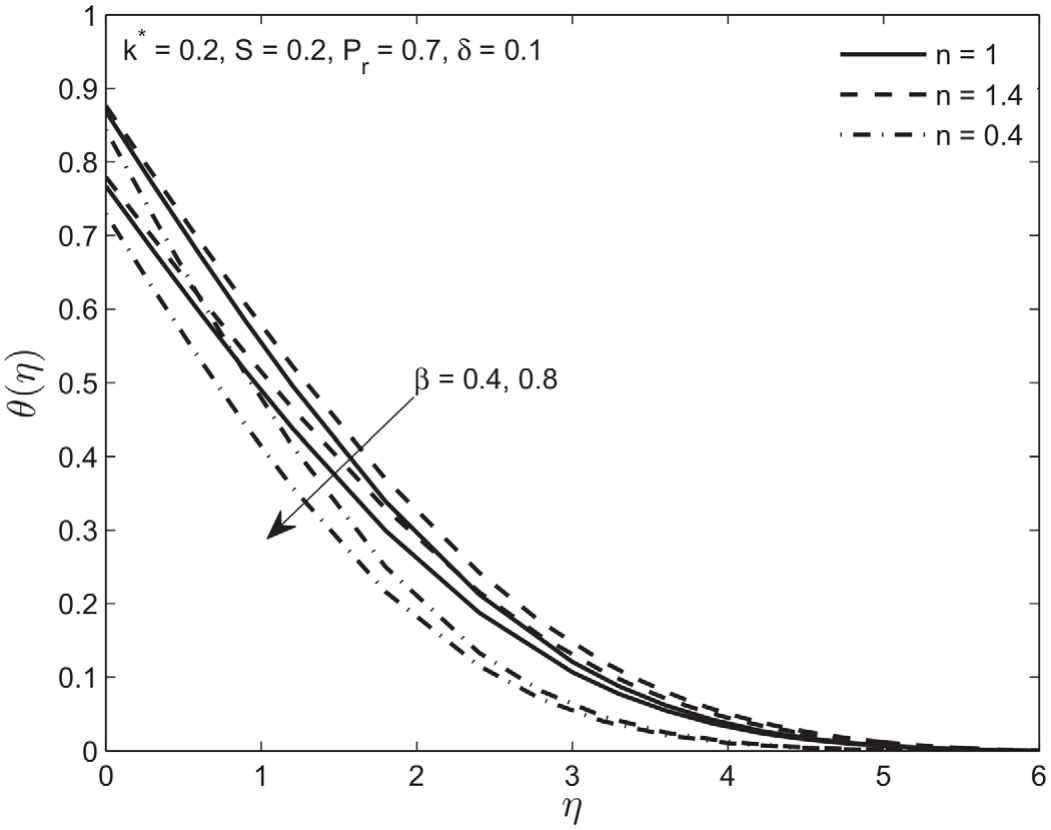
Effect of variation in temperature slip parameter *β* on temperature profile *θ*(*η*) of Newtonian and non-Newtonian fluids.

Fig 10 represents the effect of increase in temperature slip parameter *β*. It is noted that increase in thermal slip parameter decreases the temperature as less amount of heat is transferred from the embedded plate to the fluid. Again this decrease in temperature profile for the *n* > 1 case is slower and faster for *n* < 1 case as compared to the Newtonian case. The velocity profile is independent of this effect because the velocity equations are independent of the thermal slip parameter *β*.

**Fig 11:**
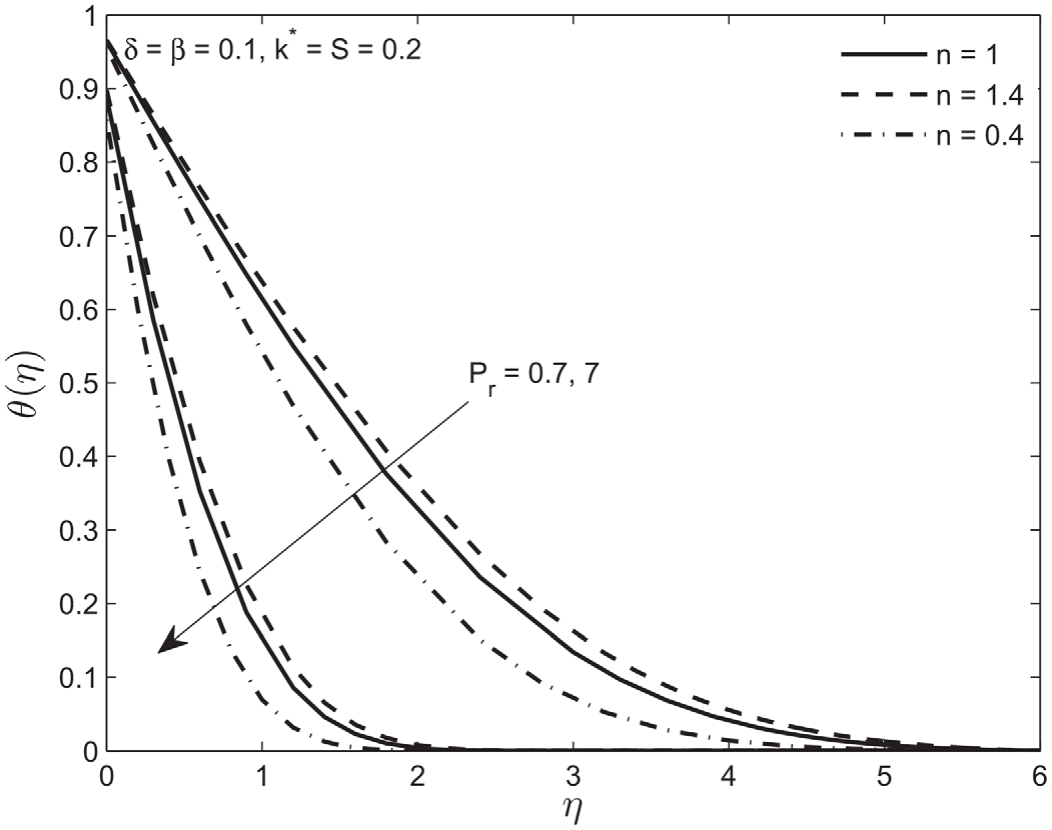
Effect of Prandtl number *P*_*r*_ on the temperature profile *θ*(*η*) of Newtonian and non-Newtonian fluids.

In Fig 11, we analyze the effect of the Prandtl number on the temperature profile. The increase in *P*_*r*_ number causes a decrease in temperature of a power law fluid at a specified distance from the porous plate. On the other hand an increase in *P*_*r*_ causes an increase in the fluid viscosity. Again this trend is slower for the shear-thickening fluid and faster for the shear-thinning fluid, as compared to a Newtonian fluid. This implies that an increase in Prandtl number is accompanied by a decrease in thermal conductivity which means that the fluid transfers more heat effectively through convection. Thereby the thickness of the thermal boundary layer gets reduced.

## Concluding Remarks and Future Work

In the present study we have conducted the study of forced convective boundary layer flow of power-law fluid along with heat transfer over a porous plate in a porous medium. The governing boundary layer equations along with the boundary conditions were first transformed to a set of coupled nonlinear ordinary differential equations using similarity transformation. The resulting system of differential equations was solved numerically. We have summarized our results based on the key parameters such as, the permeability parameter, velocity and thermal slip parameters, injection and suction parameters and Prandtl number along with variation of the power law index *n*.

The basic feature of our model could be incorporated in further studies on a variety of flow situations in complex media. The anticipation is to incorporate the additional features such as temperature dependent thermal conductivity and heat flux boundary conditions (see for example, [43–45]). The comparison can be made between the results of present simplified model and one obtained using FEM packages for the solution of boundary layer flows with slip conditions. Clearly, there is an opportunity for experimental work on these systems.
